# Interplay between Metabolism, Nutrition and Epigenetics in Shaping Brain DNA Methylation, Neural Function and Behavior

**DOI:** 10.3390/genes11070742

**Published:** 2020-07-03

**Authors:** Tommaso Pizzorusso, Paola Tognini

**Affiliations:** 1 Department NEUROFARBA, University of Florence, 50139 Florence, Italy; tommaso@in.cnr.it; 2 Department of Translational Research and New Technologies in Medicine and Surgery, University of Pisa, 56123 Pisa, Italy

**Keywords:** DNA methylation, DNA hydroxymethylation, brain, metabolism, nutrition

## Abstract

Gene expression in the brain is dramatically regulated by a variety of stimuli. While the role of neural activity has been extensively studied, less is known about the effects of metabolism and nutrition on transcriptional control mechanisms in the brain. Extracellular signals are integrated at the chromatin level through dynamic modifications of epigenetic marks, which in turn fine-tune gene transcription. In the last twenty years, it has become clear that epigenetics plays a crucial role in modulating central nervous system functions and finally behavior. Here, we will focus on the effect of metabolic signals in shaping brain DNA methylation, both during development and adulthood. We will provide an overview of maternal nutrition effects on brain methylation and behavior in offspring. In addition, the impact of different diet challenges on cytosine methylation dynamics in the adult brain will be discussed. Finally, the possible role played by the metabolic status in modulating DNA hydroxymethylation, which is particularly abundant in neural tissue, will be considered.

## 1. Introduction

### 1.1. The Nature Versus Nurture Problem: Can Epigenetics Be the Answer?

The “nature versus nurture” issue is one of the most ancient debates in neuroscience and psychology. The word “nature” refers mainly to our genes and, thus, to our genetic heritage. On the other hand, “nurture” refers to all the environmental influences, experiences and stimuli which we have been in contact with from our infancy and even during the fetal stage. The ultimate question is: how do these factors interact in shaping neural circuits and eventually our behavior? Nowadays, it is clear that the problem to address consists of understanding how environmental factors affect gene expression and how gene products can alter neural function.

Epigenetics (the study of heritable changes in gene expression that do not involve changes to the underlying DNA sequence [1]) can be considered a sort of bridge between genes and environment, and a new regulatory platform to integrate information coming from experience into stable modifications in behavioral outcomes. Thus, epigenetics is the key that has finally disentangled the “nature versus nurture” diatribe. 

The last twenty years have witnessed challenging efforts to identify epigenetic mechanisms both in health and disease: histone post-translational modifications (PTMS), DNA methylation, non-coding RNA and nucleosome remodeling. Among several epigenetic mechanisms used by the cell, DNA methylation, catalyzed by DNA methyltransferases (DNMTs), has been deeply investigated as a key regulator of transcriptional silencing. For instance, it plays a paramount role in several cellular processes such as regulation of gene expression, silencing of transposable elements, genomic imprinting and X chromosome inactivation [2]. Methylation occurs at position 5’ of cytosine (5-mC), mostly located in CG dinucleotide (CpG); however, non-CG methylation (mCH) has been recently discovered in the adult brain at a high level [3,4] and in virtually all the human tissues [5]. 

This review will describe how metabolic signals, prevalently derived by nutritional changes, affect DNA methylation levels in the brain during development and adulthood. The possibility that metabolism could modulate brain 5-hydroxymethylcytosine (5-hmC) will be also discussed. Finally, open questions and future approaches to potentiate the causal connection between neuroepigenetics and metabolism will be examined.

### 1.2. Dynamic DNA Methylation in the Brain: An Overview 

DNA methylation was long considered a stable epigenetic mark in post-mitotic cells, such as neurons. However, despite their necessity to maintain genomic stability, neurons continuously integrate endogenous and exogenous signals, often resulting in plastic mechanisms at different levels (molecular, structural and functional). The epigenome represents a platform able to process environmental stimuli, translating them into plasticity, and giving the neuronal cells the opportunity to switch from a poised/quiescent state to an active state toward circuit modifications.

In the last decade, a plethora of studies demonstrated how brain DNA methylation is highly modulated by activity and experience during prenatal and postnatal development, and adulthood, challenging the traditional view of a stable epigenetic mark [6]. For instance, in one of the first studies, electroconvulsive stimulation of the adult mouse brain was found to induce rapid modifications in the CpG methylome of the dentate gyrus. Interestingly, the majority of the changes in activity-dependent DNA methylation were enriched in intergenic regions and were not necessarily correlated to gene expression, suggesting a major complexity in cytosine methylation-dependent transcriptional control in dentate gyrus cells [7]. Gene-specific and whole genome modifications in DNA methylation in the brain were observed in response to learning and memory processes [8,9,10,11,12,13,14], drug addiction [15,16,17,18], stress and physical exercise [19,20,21]. Furthermore, experience-dependent changes in 5-mC on the promoter of plasticity-related genes, such as microRNA 132 [22,23], regulated visual cortical plasticity during postnatal development [24]. Finally, activity-dependent processes sculpting neuronal circuits during postnatal development remodel cytosine methylation, and in particular, non-CG methylation accumulates in neuronal cells and becomes predominant during adulthood [25]. As there are several excellent reviews on the above-mentioned arguments [26,27,28,29,30], we will not discuss those topics deeply. We will, instead, concentrate on less explored factors capable of modulating brain DNA methylation: metabolism and nutrition.

Environmental stimuli including diet, physical exercise and stress often converge into changes in the metabolic status of the individual. Metabolism impinges on whole-body physiology and health, including the central nervous system (CNS). This happens in part through the modulation of epigenetic processes. A historical example is represented by periconceptual exposure to the Dutch Hunger Winter (1944–45), which caused an increase in DNA methylation of the imprinted *IGF2* gene still present six decades later [31]. The agouti mouse is another classic model explaining how maternal diet derived micronutrients can affect epigenetic regulation, gene expression and subsequently the phenotype. Indeed, the viable yellow agouti (*A^vy^*) mouse model, in which coat color variation is correlated to epigenetic marks established early in development, has been used to investigate the impact of nutritional and environmental influences on the fetal epigenome. This mouse results from the insertion of an intracisternal A particle (IAP) murine retrotransposon upstream of the transcription start site of the *Agouti* gene. DNA methylation of the IAP inversely correlates with the level of ectopic *Agouti* expression and causes a wide variation in coat color ranging from yellow (unmethylated) to light brown (methylated) [32]. 

In the next sections we will focus on nutritional challenge-driven changes in DNA methylation during brain development and in adulthood and the subsequent effect on neural function and cognitive and emotional outcomes. 

### 1.3. Diet and Metabolism Influence on DNA Methylation in the Developing Brain 

In the last decade, a growing body of studies has linked nutrition to epigenetics since the chemical moieties used for PTMS are often donated by metabolites [33]. Indeed, S-adenosylmethionine (SAM), the chemical donor for DNA methylation and other methylation-involving reactions, derives from a biochemical cascade called one-carbon metabolism [34].

**Figure 1:**
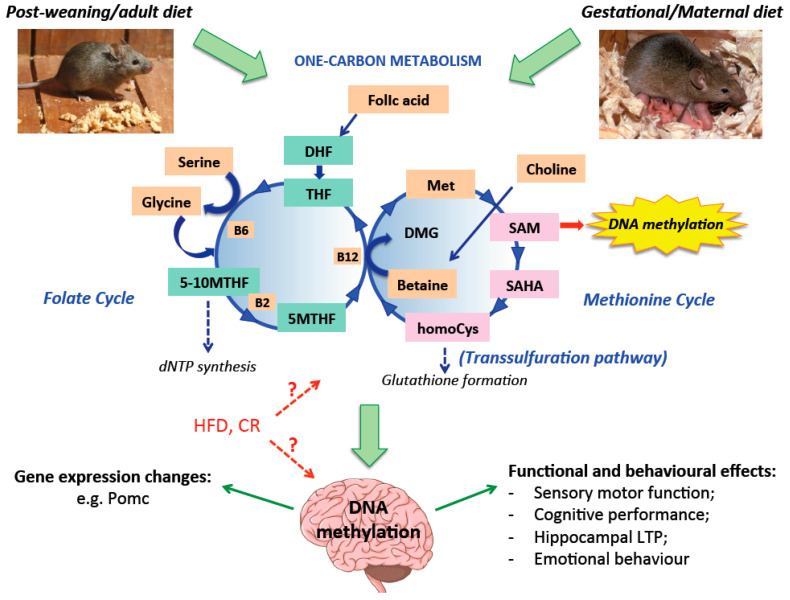
Nutrition affects one-carbon metabolism and DNA methylation. Gestational/maternal diet before weaning influences DNA methylation in the brain of the offspring. In addition, life-long nutrition and the metabolic status impact on brain DNA methylation. Micronutrients from the food could impinge on one-carbon metabolism: molecules introduced in our body with the diet are reported in orange squares; vitamins are also reported. Those molecules enter in the folate cycle (left) or the methionine cycle (right), possibly changing the availability of SAM, the universal methyl group donor used by the SAM-dependent methyltransferase enzyme for DNA (or other substrates) methylation reactions. Modification in the pattern of DNA methylation in specific genomic loci in the brain could consequently modulate gene expression and behavioral outcome. Acronym: met = methionine; SAM = S-adenosylmethionine; SAHA = S-adenosylhomocysteine; homoCys = homocysteine; DMG = dimethylglycine; DHF = dihydrofolic acid; THF = tetrahydrofolic acid; 5MTHF = 5-methyl-tetrahydrofolate; 5-10MTHF = 5-10 methylenetetrahydrofolate; B12 = vitamin B12, precursor to methionine synthase, involved in the production of met from homoCys and betaine; B6 = vitamin B6, cofactor in the conversion of THF to 5-10MTHF; B2 = vitamin B2 involved in the conversion of 5-10MTHF to 5MTHF; HFD = high fat diet; CR = caloric restriction; LTP = long term potentiation. Color coding: green squares = compound belonging to the folate cycle; pink squares = compound belonging to the methionine cycle; orange squares = micronutrient derived from the diet. Blue dashed arrows = omitted intermediate reactions; red dashed arrows = involvement not demonstrated yet.

One-carbon metabolism integrates information coming from the metabolic status of the body into three different biochemical pathways: the folate cycle, the methionine cycle and the transsulfuration pathway (Figure 1). The methionine cycle begins with homocysteine that accepts the carbon from the folate pool through the 5-methyltetrahydrofolate to generate methionine. Methionine, through methionine adenyltransferase, is used to generate SAM, which is demethylated to form S-adenosylhomocysteine (SAHA). After deadenylation, SAHA is converted back to homocysteine, resulting in a full turn of the methionine cycle [35].

As methionine is introduced with the diet, it is easy to theorize that alterations in the nutritional level of methionine could impact on SAM production and, as a consequence of that, on the level of DNA methylation. However, other micronutrients present in the food can contribute to methyl group production, such as folate and choline, as they participate in one-carbon metabolism as precursors. Notably, choline is also required for the synthesis of phospholipids, transmembrane signaling, lipid-cholesterol transport and cholinergic neurotransmission [36]. This metabolic influence is thought to be present at all ages, already beginning during prenatal life when the epigenome shows the most dramatic remodeling [37].

It is worth noting that during gestation the nutrition of the fetus relies completely on maternal diet. DNA methylation in the fetal brain has been shown to be present and localized on specific genomic regions in both humans and mice [25,38]. Thus, the fetal brain DNA methylome and its configuration immediately after birth must be, at least in part, affected by the surrounding nutritional milieu. Although there are no systematic analyses of the changes in the cerebral cytosine methylome at different stages of life related to the maternal diet, several reports have demonstrated how modifications in the availability of micronutrients working as methyl group donors and cofactors could influence brain function and behavior in the offspring. For instance, a mix of methyl donor supplements during gestational and preweaning periods was able to alter neurodevelopmental trajectories in C58 mice, ameliorating repetitive motor behavior and increasing global DNA methylation in the cortex and cerebellum in the adult offspring [39]. Furthermore, changes in folic acid supplementation in maternal diet resulted in altered CG and CH methylation at promoters and gene bodies in the cerebral cortex of the offspring [40]. Folic acid supplementation in the maternal diet during the second and third gestational week or during the whole pregnancy significantly increased folate concentration in the brain of rat offspring in correlation with a decrease in global DNA methylation. The effect was tissue specific, since no changes in DNA methylation were observed in liver, kidney or colon, suggesting the neural tissue to be particularly sensitive to folate administration. However, no alterations in specific gene expression, no DNA methylome or locus specific analysis of 5-mC were reported [41], making necessary a more precise investigation. Moreover, periconceptual folate administration improved sensory motor function in the offspring together with increased global 5-mC and activity and expression of DNMTs [42]. In contrast, excessive folate during pregnancy was found to be deleterious, causing short-term memory impairment, decreased hippocampal size, decreased thickness of the dentate gyrus and altered expression of one-carbon metabolism enzymes in the offspring. Moreover, *Dnmt3a* expression was lower in both the cortex and hippocampus, suggesting the possibility of epigenetic alterations underlying the observed phenotype [43]. High folate during gestation was also obesogenic in rodent offspring through alteration in the level of hypothalamic genes implicated in the control of feeding behavior, concurrent to DNA methylation changes on POMC (pro-opiomelanocortin) and insulin receptor promoters [44]. 

It is worth noting that paternal dietary methyl donor intake also influences behavioral and cognitive functions in the next generation. The offspring of fathers fed a methyl donor-enriched diet (folic acid, L-methionine, choline, zinc, betaine and vitamin B12) displayed deficits in contextual fear conditioning and spatial learning associated with impaired hippocampal long-term potentiation (LTP) and electroencephalogram oscillatory pattern in the 5–10 Hz range (theta oscillations). Gene expression analysis revealed a downregulation of the BK channel subunit *Kcnmb2* and hypermethylation of the same gene. Overexpression of *Kcnmb2* in the offspring was able to rescue the phenotype and hippocampal dysfunction [45,46]. On the other hand, paternal dietary methyl donor depletion elicited greater depression-like behavior in the forced swim test and increased anxiety-like behavior in the open field in rat offspring; however, no molecular mechanisms were explored in this context [47]. In addition, in paternal folate deficiency models, the percentage of 5-mC and protein expression of IGF-2 in the fetal whole brain were lower [48,49]. Those studies suggest that methyl donors and one-carbon metabolism could impact on embryonic and fetal brain function, not only directly, through the exchange of nutrients with the mother during the gestational period, but also indirectly through paternal epigenetic inheritance influencing the germline. 

Epidemiological and preclinical studies report that exposure to maternal obesity or high fat diet (HFD) during gestation increases the risk of neurodevelopmental disorders [50,51,52]. Interestingly, methyl donors have been demonstrated to rescue negative behavioral outcomes driven by in utero exposure to HFD. The mouse offspring of mothers fed HFD, to mimic maternal obesity, displayed increased cysteine in the prefrontal cortex consistent with oxidative stress and a dramatic decrease in DNA methylation exclusively in males. Methyl donor supplementation to the male offspring after weaning was able to restore the prefrontal cortex DNA methylation levels and to restore the concentration of several one-carbon metabolism intermediates [53]. Moreover, this dietary supplementation strategy was able to rescue female offspring impairment in prefrontal cortex-mediated executive functions [54]. Interestingly, addition of methyl donors to maternal HFD attenuated the development of some of the adverse effects including weight gain, increased fat preference and global hypomethylation in the prefrontal cortex [55]. Folate supplementation to HFD-fed mothers rescued weight gain, cognitive performance and anxiety-like behavior in the offspring. Finally, changes in neurodevelopmental-related gene levels and corresponding DNA methylation were normalized to control by maternal folate consumption [48]. 

All together these findings confirmed that micronutrient availability fine tunes DNA methylation in the offspring brain and that subtle alterations in the balance of specific metabolites can have dramatic effects on the chromatin landscape of neural tissue, with severe behavioural outcomes. Lastly, aberrant epigenetic remodeling driven by a food challenge could be normalized by specific micronutrient supplementation. These observations open questions about how epigenetic enzymes sense metabolic cofactor availability and how plastic 5-mC acquired during gestational epigenetic programming could be. Moreover, further studies are needed to dissect alterations in the whole brain methylome in different CNS regions and even at the level of specific neuronal cell types.

Although we lack a profound overview of the maternal diet-dependent neural tissue DNA methylome, some genes seem to be exquisitely impacted by changes in 5-mC. For instance, the *Pomc* gene locus has been shown to be a target of the epigenetic remodeling driven by maternal diet. POMC-expressing neurons are enriched in the arcuate nucleus of the hypothalamus, and they are critical regulators of metabolism, energy balance (reducing food intake and increasing energy expenditure) and reproduction [56]. It is known that maternal HFD alters offspring feeding behavior, often leading to obesity. This phenotype is in part mediated by changes in the concentration of hypothalamic orexigenic peptide POMC, driven by alteration in DNA methylation [57]. Offspring of HFD-fed mothers displayed long-lasting increases in *Pomc* promoter methylation and body weight, even upon consumption of a balanced/standard diet (regular chow with 6% of Kcal from fat), after weaning [58]. In a similar study, maternal HFD re-programmed the epigenetic landscape of *Pomc* promoter and enhancer regions causing hypermethylation in the arcuate nucleus of rats weaned on a low-fat diet [59]. It seems that not only 5-mC but also 5-hmC is implicated in the regulation of *Pomc* transcription in the arcuate nucleus. The *Pomc* promoter of balanced diet-fed rats exposed to maternal HFD had increased 5-mC, while one of the offspring from balanced chow-fed mothers displayed enrichment in 5-hmC. Importantly, there were negative correlations in the body weight of individual rats and their 5-hmC levels. In contrast, 5-mC levels were positively correlated with body weight [60]. On the other hand, mouse offspring from maternal high fat, high sucrose diet and consuming the same food after weaning exhibited *Pomc* promoter hypomethylation and increased *Pomc* expression in the hypothalamus, together with increased body weight and decreased glucose tolerance and insulin sensitivity [61]. The discordance among the reports could be due to different rodent models (rats vs. mice) and by the presence of high sucrose in the chow consumed in the study by Zheng et al.; moreover, the ratio in the various macro- and micronutrients should also be taken into account, not only for the metabolic but also for the epigenetic effect. Nowadays, it is not clear which molecular mechanisms are responsible for the modulation in the DNA methylation of *Pomc* or other gene loci in response to maternal HFD feeding. 

As previously mentioned, choline is an important donor of methyl groups. This metabolite is oxidized to betaine, which is a substrate in the betaine-homocysteine methyltransferase reaction, which links choline and betaine to folate-dependent one-carbon metabolism [62]. Remarkably, there is usually a direct relationship between dietary intake of choline and tissue levels of SAM [63,64]. Choline demand is higher during pregnancy, and choline supplementation during gestation and the perinatal period have been shown to be neuroprotective and to improve cognitive performance in adulthood [65]. For instance, choline gestational deficiency caused a decrease in global DNA methylation in the Ammon’s horn ventricular and subventricular zones, whereas no significant changes were observed in the dentate gyrus in the fetal brain [66]. Moreover, a diet deficient in methyl groups, such as choline, may result in altered DNA methylation, not only in the brain but in a variety of tissues, resulting in modified offspring phenotype, especially in relation to metabolic homeostasis and carcinogenesis [67]. Choline administration immediately after birth was able to normalize global DNA methylation levels in the cortex and hippocampus of rodent pups subjected to postnatal alcohol exposure [68]. Similar effects were observed in the offspring of mothers treated with alcohol during gestation. Choline supplementation simultaneously to alcohol exposure reversed the DNA hypermethylation and deregulation in hypothalamic *Pomc* gene expression and attenuated stress hyperresponsiveness during adulthood [69]. 

Lastly, it is well known that inadequate levels of folate during pregnancy could increase the risk of developing neural tube defects (NTD) in the baby. How folate supplementation could avoid NTD is not known; nonetheless, some studies have suggested that altered patterns of DNA methylation in humans could be involved [70]. HPLC analysis of 5-mC in skin, heart, lung, brain, kidney and liver tissues demonstrated aberrant levels in human NTD fetuses with respect to controls and significant hypomethylation in the brain of NTD fetuses [71]. Investigations in a mouse model of NTD characterized by folate dysmetabolism obtained through methotrexate injection in pregnant mice, showed that embryonic neural tube tissue had significantly decreased global DNA methylation levels compared to control. Genes belonging to the Wnt pathway (*Siah1b*, *Prkx*) displayed genomic loci hypomethylation and subsequent upregulation in their levels. Those genes might be relevant players in this pathology, as the Wnt pathway is an important regulator of neural tube development [72]. Despite this preliminary correlative information, we still lack causal and mechanistic proof to link DNA methylation, modification in the expression of genes important for embryonic development and NTD. 

In summary, these lines of evidence indicate that DNA methylation may respond to the supply of methyl groups in a complex fashion that includes alterations in the activities of DNA methylating and/or demethylating enzymes. For example, diet can influence the expression of DNMTs [42,73,74]. It was demonstrated that the DNA methylation signature acquired during development can be transferred through cell divisions thanks to DNMT1, an enzyme that methylates hemimethylated CpG loci and allows the parental methylation pattern to be conserved. On the other hand, the de novo DNMTs, DNMT3A and DNMT3B establish new patterns of methylation and show the same activity toward unmethylated or hemimethylated DNA [75]. Although in postmitotic neurons the function of DNMTs might be different, the two categories of DNMTs might concur to rapidly respond to environmental inputs and experiences. It is worth noting that not all the CG sites are sensitive in the same manner to methyl group availability, suggesting a certain degree of specificity not yet understood. Moreover, DNMT enzymes and demethylation proteins expression and activity could be modulated by diet composition and probably also their chromatin recruitment, complicating the regulation of DNA methylation. Finally, the brain is characterized by an intrinsic complexity due to distinct functional areas, layers and cell types, further entangling the investigation of DNA methylation dynamics. 

### 1.4. Impact of Nutrition on Methylation of DNA in the Adult Brain

Despite the decrease in plasticity observed after sensitive periods of postnatal development, the adult brain still experiences plastic processes at molecular, anatomical and functional levels. Epigenetic marks dynamically react to external signals and can be modified based on the availability of cofactors and metabolites all life long. Nutritional challenges can modulate DNA methylation in the adult CNS with relevant consequences in gene expression, physiology and behavior. 

Feeding post-weaning mice a folate, methionine and choline deficient chow impaired hippocampus-dependent memory and caused promoter hypermethylation and low expression of specific subunits of AMPA (α-amino-3-hydroxy-5-methyl-4-isoxazolepropionic acid) receptors [76]. A diet rich in methionine and low in vitamins B6/B12 led to altered memory function in the passive avoidance test, increase in global 5-mC, specific hypermethylation of the *Netrin1* promoter and downregulation of *Netrin1* gene expression. Importantly, intracerebral administration of *Netrin1* considerably restored long-term fear-motivated memory [77]. Methyl donor supplementation later in life has been demonstrated to rescue depression-like behavior induced by maternal separation and to increase global brain DNA methylation in adult rats [78]. 

As maternal HFD was shown to impact offspring brain DNA methylation, feeding a Western diet to adult mice for 12 weeks caused a decrease in global DNA methylation in the frontal cortex. Genome-wide bisulfite sequencing identified differentially methylated loci belonging to genes related to neurological function and disease, neuroactive ligand-receptor interaction, axon guidance, regulation of metabolism, cell adhesion, leukocyte transendothelial migration and inflammation [79]. This is one of the few articles in which a genome-wide DNA methylome analysis was performed in a specific brain area in response to a food stress. The authors speculate that changes in DNA methylation could potentially provide an explanation for how a Western diet impacts cognitive performance and dementia; however, further work would be necessary to provide a causative link between diet, DNA methylation and cognitive status. Furthermore, HFD had relevant effects on the striatum, where it downregulated the dopamine D2 receptor (D2R) via an increase in DNA methylation on its promoter. Notably, administration of γ-oryzanol, the bioactive constituent of brown rice, decreased the expression and activity of DNMTs, thereby restoring the level of D2R in the striatum [80]. Therefore, γ-oryzanol might be an interesting molecule to counteract epigenetic misregulation dependent on HFD consumption, potentially ameliorating metabolic and cognitive dysfunctions. 

Diet-induced obesity impaired hippocampal performance in spatial memory, synaptic plasticity tested through LTP and decreased the levels of memory related genes (*Ppp1cb*, *Reln*, *Sirt1*) in the hippocampus. Intriguingly, all the promoters of the memory-related genes displayed hypermethylation. Neuron-specific Sirt1 knockout (KO) mice recapitulated deficits in hippocampal memory, while resveratrol treatment rescued them in obese mice [81]. Cafeteria diet (obtained by adding to the standard chow also French fries, parmesan cheese, cheese flavored snacks, crackers, cookies, pudding, peanut butter and chocolate; food items chosen to reflect the enormous variety, palatability and energy density of the modern Western diet) altered the expression of hormone receptors and steroidogenic enzymes in specific hypothalamic nuclei of rat females. DNA methylation was decreased in distinct loci of *Npy* and *Pomc* promoter regions in the paraventricular nucleus and the arcuate nucleus, respectively. The epigenetic regulation of gene expression was correlated with changes in energy intake, body weight and fat depots, suggesting that cafeteria diet alteration of 5-mC hypothalamic nuclei could be responsible for long-lasting modifications in metabolic homeostasis [82]. 

Alcohol, an abuse substance commonly drunk in a social context, is often part of the diet in adult individuals. Its moderate to heavy consumption is known to deteriorate cognitive abilities and to induce neural damage over time. The specific mechanisms are still under exploration; however, metabolites generated upon alcohol metabolism might contribute through epigenetic remodeling and long-term modulation of transcription [83]. As such, alcohol exposure not only programmed DNA methylation in the fetal brain [84], but also impacted one-carbon metabolism in the adult. Indeed, chronic alcohol exposure decreased SAH levels and increased the SAM/SAH ratio and the expression of *Mat2a* and S-adenosyl homocysteine hydrolase (*Ahyc*) in rat cerebellum. 5-mC was not analyzed, although the authors expected brain hypermethylation based on their data [85]. 

With increased life expectancy, healthy aging has become an important matter in the scientific field, and the CNS has definitely been in the spotlight, since brain aging is often accompanied by an increased risk of cognitive decline and neurodegenerative diseases [86]. Intriguingly, DNA methylation has been linked to aging-driven modifications in gene transcription [87]. As nutrition and aging are profoundly intertwined, diet manipulations have been demonstrated to modulate DNA methylation in the aging brain. Caloric restriction (CR) improves cognitive performance, impacts neurogenesis and is neuroprotective [88]. A recent study showed that life-long CR in mice prevented age-related differential CG and CH methylation in the hippocampus. The effect was specific for genomic elements connected to fundamental aging processes, such as neuronal cell structure and integrity and cell metabolic pathways. Notably, CR was able to induce a de novo pattern of CG and CH methylation in the aging brain, impacting genes involved in neuroprotection, probably through modulation of epigenetic enzymes such as the ten eleven translocation enzyme (TET) 2 and DNMT3A1 [89]. CR was also able to prevent 5-mC immunoreactivity in the hippocampus and cerebellar Purkinje cells of old mice; however, these studies did not analyze the methylation profile of genes, precluding any conclusion about the functional relevance of the observed results [90,91]. 

The investigations performed both during development and adulthood convincingly indicate the existence of a tight link between metabolic changes and DNA methylation. However, more exploration is needed to identify, on a genome-wide scale, which chromatin regions are affected, which specific genes are modulated and, on top of that, how those epigenetic/molecular modifications are causally related to alterations in neural function and finally behavior. 

Reprogramming of 5-mC driven by HFD has been observed in different tissues, thus suggesting that methylation plays an important role in regulating the response to diets rich in fat. Changes in the level of one-carbon metabolism components, such as SAM and SAHA, could be taken into account. For example, hepatic SAM and SAHA gained diurnal oscillation in their level in adult HFD-fed mice together with some transcripts coding for the enzymes related to the same biochemical pathway [92]. In the prefrontal cortex and suprachiasmatic nucleus (SCN) of HFD-fed mice SAM was not significantly altered, although methionine was decreased, while SAHA was significantly decreased only in the SCN [93], indicating that modulation of one-carbon metabolism intermediates could explain part of the methylation effects observed. Furthermore, folate depletion in maternal diet followed by post-weaning HFD, caused downregulation of the base excision repair (BER) pathway involved in DNA demethylation [94,95]. BER reduction was present in the offspring cortex, hippocampus and subcortical regions. The effect was accompanied by an increase of 5-mC and a significantly lower 5-hmC/5-mC ratio in the cortex [96]. Thus, alteration in BER enzyme expression and/or activity could be another mechanism to explain HFD-driven modifications in the methylation of DNA. However, so far no direct (impact on one-carbon metabolism micronutrient levels) or indirect (changes in gene expression) proved molecular mechanisms linking HFD or obesity to altered 5-mC or 5-hmC in the brain have been discovered.

CR effects on DNA methylation in different tissues is of much interest in light of the new concept of epigenetic clocks, which measures changes in CpG sites to predict chronological age [97]. CR has been shown to promote longevity, and its impact on 5-mC could contribute to this effect. The mechanisms involved are not unveiled yet. A decrease in DNMT3A expression has been observed in the hippocampus of aged mice upon CR [98]. In the liver, hypomethylation of enhancers was decreased by CR and rapamycin [99,100], suggesting that the mTOR pathway might play a role; however, no specific epigenetic mechanisms have been explored. 

It is worth noting that tissue- and cell-specific analysis is required to discover how nutrition affects DNA methylation and other epigenetic marks, especially in the brain. It could be likely that, based on their function, brain regions will not be equally affected by metabolic alterations. Importantly, as the neural tissue is so heterogeneous, the distinct cell types might respond in different ways and their chromatin landscape could be more or less resilient to the surrounding metabolic milieu. For example, inhibitory interneurons in the human prefrontal cortex displayed high levels of 5-hmC at specific enhancers as compared with glutamatergic neurons. In gene bodies, the range of 5-hmC levels between genes with low and high expression was also significantly higher in inhibitory than in excitatory neurons, with better correlation of DNA hydroxymethylation with transcription in GABAergic neurons [101]. Thus, cell-specific analysis will avoid the ambiguity of measurements on mixed cell types, defining a precise profile in DNA methylome and hydroxymethylome upon metabolic challenges.

Finally, the gut microbiota is strongly intertwined to the host metabolism and might be another, so far underestimated, player in the topic of metabolism-driven neuroepigenetics. The gut microbiota are involved in immune system function [102], metabolic homeostasis [103], circadian rhythms [104,105] and even CNS function and behavior [106]. Intriguingly, the intestinal microbiome work as an endogenous factory for producing complementary endogenous sources of, among others, vitamin B12, B6 and folate; also microbes contribute to energy harvesting from food and nutrient absorption [107]. Thus, the microbiota could potentially modulate the availability of chemical donors for DNA methylation. Recently, it was demonstrated that microbiota mediate changes in DNA methylation of intestinal epithelial cells through a mechanism involving TET2/3 activity [108]. Moreover, the bacteria are implicated in controlling TLR4 gene (which activates the innate immune system) methylation in mouse intestinal cells [109]. Those effects were observed locally and were not linked to micronutrients/diet. A methionine-restricted diet modulated the composition of the gut microbiota in a sex-dependent manner. DNA and protein methylation were analyzed only in the liver, and no changes were found, excluding both a direct diet impact and an indirect one from the microbiota [110]. 

Microbial-derived short-chain fatty acids (SCFA, acetate, butyrate and propionate), obtained through the fermentation of indigestible polysaccharide, work as histone deacetylase inhibitors (HDAC) and have been shown to cross the blood‒brain barrier. SCFA contribute to maturation and maintenance of microglial cells function [111] and may modulate the levels of neurotransmitter and neurotrophic factors, and altered levels of SCFA have been observed in several brain disorders [112]. Furthermore, SCFA seem to influence DNA methylation in colon cancer cells [113] 

At the moment, there is no direct evidence demonstrating that the intestinal commensals could influence epigenetics in the CNS. Notwithstanding, the gut-microbiota-brain axis has been involved in neurophysiology and pathology at different levels; therefore, we cannot exclude that microbes-derived metabolites could reach the brain and impact on the epigenetic landscape of its cells. 

### 1.5. Can the Metabolic Status Influence DNA Hydroxymethylation?

The above-mentioned studies and many more demonstrate the dynamic nature of cytosine methylation and its reversibility in the neural tissue in response to metabolic challenges. 

How do 5-mC dynamic changes occur? A single enzyme capable of breaking the strong carbon‒carbon bond in order to directly demethylate cytosine has not been found yet. However, cytosine demethylation can occur through a series of chemical reactions of deamination and/or oxidation catalyzed by specific enzymes. Among the enzymes, growth arrest and DNA damage-inducible 45 proteins (GADD45A and GADD45B) have been shown to take part in the demethylation process in the brain [114,115]. A major breakthrough for the understanding of DNA demethylation, and consequently its modifiability, was the discovery of TET proteins and the capability of TET1 to convert 5-mC to 5-hmC [116]. A few years later, TET2 and TET3 were also identified to have the same enzymatic properties of TET1 [117]. 

5-hmC immediately raised neuroscientists’ curiosity as quantitation experiments have revealed this mark to be remarkably abundant in the CNS with respect to other organs [118], perhaps indicating that it is not only a biochemical reaction intermediate. Furthermore, there were differences in 5-hmC abundance in the neural tissue: neuronal progenitors, young neurons and adult neural stem cells had low levels of 5-hmC [119,120,121]. Thus, 5-hmC seems to be an epigenetic mark belonging to well-differentiated postmitotic neurons, consistent with its quick upregulation during synaptogenesis and neuronal development in the postnatal brain [119,121]. In contrast to 5-mC, 5-hmC has been correlated with active transcription and is prominently localized at the gene bodies of actively transcribed genes [25,122,123]. 

As TET enzymes belong to the family of 2-oxoglutarate-dependent dioxygenases, which needs Fe^2+^, 2-oxoglutarate/α-ketoglutarate (AKG) and reducing agents, such as ascorbic acid (i.e., vitamin C), to catalyze chemical reactions [124], the metabolic status might contribute to regulate DNA hydroxymethylation. 

A recent article reported that CR could prevent alterations in 5-hmC levels observed in the hippocampus in old mice [125]; a similar effect was observed by the same group with global DNA methylation [91]. In contrast, CR was not as effective in preventing age-dependent increase in 5-hmC in cerebellar Purkinje cells [90]. As already described, lean offspring of non-HFD-fed mothers resulted in a reduction of 5-mC and an increase of 5-hmC within the promoter of *Pomc* in the hypothalamus [60], and diet-induced obesity caused DNA hypo-hydroxymethylation of the *Sirt1* promoter with consequences in gene expression and memory performance [81]. 

Despite sparse information in a few studies, there is no direct evidence connecting 5-hmC to the metabolic status of the individual. However, it is possible to speculate that changes in the level of ascorbic acid in the diet could affect TET activity and finally 5-hmC. Strikingly, vitamin C has the highest concentration of the whole body in the brain, where it is actively transported via the sodium-dependent vitamin C transporter SVCT-2 [126]. Moreover, ascorbic acid is increased during development in the rodent brain [127] as well as 5-hmC. In KO mice for the ascorbic acid biosynthetic enzyme (to mimic the human condition that has no vitamin C endogenous biosynthesis), the levels of 5-hmC were several times higher with respect to liver or lung after ascorbic acid supplementation. The withdrawal of ascorbic acid from the diet significantly decreased total 5-hmC brain concentration suggesting that this epigenetic mark is sensitive to ascorbic acid [128]. Moreover, vitamin C treatment of astrocytes in vitro promoted their differentiation, in association with modification in DNA hydroxymethylation [129]. 

AKG is a Krebs cycle intermediate synthesized by IDH1/2 enzymes. As such, it regulates anabolic and catabolic Krebs cycle products and substrates, thereby regulating amino acid synthesis, ATP production and reducing equivalent (NAD^+^/NADH) generation, which in turn could influence reactive oxygen species levels [130]. Indeed, AKG has a variety of physiological functions, from modulation of protein synthesis and bone development to maintenance of immune system homeostasis [131] and even aging [132]. Recently, it was reported that AKG dietary supplementation promoted beige adipogenesis and prevented obesity in mice [133]. Notably, its availability has been shown to be necessary for proper TET functions, since IDH1 mutations in human glioma and accumulation of an aberrant metabolite, 2-hydroxyglutarate, which is a competitive inhibitor of AKG-dependent dioxygenases, inhibited TET activity and decreased 5-hmC [134]. 

Although the possible relationship between AKG levels and neural DNA hydroxymethylation has not been explored yet, dietary AKG supplementation might contribute to modulate 5-hmC and in turn dynamic DNA demethylation in neuronal cells. 

In summary, there are no systematic studies demonstrating that nutrition and/or metabolism can affect DNA hydroxymethylation in the brain. The literature might suggest this possibility, however, future investigations are necessary to understand how diet and/or specific supplements could influence 5-hmC with subsequent impact on neural physiology. This might be relevant since vitamin C is commonly used and often recommended to children, adults and the elderly and AKG is commercially available to ameliorate sport performance. 

## 2. Summary and Outstanding Questions 

The intricate relationship between epigenetics and metabolism has been emerging in several fields, including neuroscience. The brain is sensitive to the metabolic status, as shown by behavioral and functional alterations after malnourishment [135], different types of diet [ketogenic diets (very low carbohydrate, high fat, adequate protein diet), HFD, CR [136], physical exercise and stress [137]. Several studies tried to reveal the connection between nutritional challenges and DNA methylation in the brain during development, adulthood and aging (Figure 1). Despite accumulating evidence on this matter, the results are often correlative and further exploration is necessary to causally link macronutrient/micronutrient changes to DNA methylation, gene expression control and lastly neural physiology and behavioral outcome. Moreover, there is a lack of understanding regarding the molecular mechanisms through which the diet regimen could impinge on epigenetic modifications of the neural chromatin landscape. How DNA methylation could regulate transcription in response to the metabolic status is still obscure, as well as the whole epigenetics context and cross-talk with other PTMS, which also depend on metabolism derived chemical donors (e.g., histone methylation and acetylation). 

Thus, several questions still need an answer: what are the tissue-specific mechanisms responsible for diet-driven alterations in DNA methylation? In which brain areas is DNA methylation more sensitive to micronutrient supplementation/deficiency and how this can be connected to changes in brain function and behavior? Is DNA methylation in distinct brain cell types impacted in the same way by diet and micronutrient availability? Can we use the cell-specific or area-specific diet/micronutrient-driven DNA methylome to predict neurological disease or behavioral outcomes during development, adulthood or aging? Could the gut microbiota play a role in shaping CNS DNA methylation through specific, still unknown signaling molecules or microbe-derived metabolites?

A deeper exploration at the level of distinct areas and/or cells will help to comprehend the specific molecular/epigenetic mechanisms, which could be clinically relevant for the treatment and prevention of neuropsychiatric disorders or metabolic diseases.
